# Parents’ Perceptions of Changes in Sleep Duration, Physical Activity, and Sedentary Behavior in Arab Israeli Children during the COVID-19 Outbreak

**DOI:** 10.3390/ijerph20116041

**Published:** 2023-06-02

**Authors:** Rafat Ghanamah, Hazar Eghbaria-Ghanamah, Nabil Abu-Saleh, Sujood Kitany

**Affiliations:** 1 Early Childhood Education Department, Oranim Academic College of Education, Kiryat Tevo’n 3600600, Israel; 2 Israel Ministry of Education, Jerusalem 91911, Israel; 3 Clalit Health Services, Sakhneen 3081000, Israel

**Keywords:** Arab Israeli children, COVID-19, physical activity, sedentary behavior, sleep duration

## Abstract

The COVID-19 pandemic led many countries to apply lockdown measures that could prevent children from achieving the physical activity, sedentary behavior, and sleep levels suggested for their psychophysical health. The current study tested changes in physical activity, sedentary behavior, and sleep length of children and the incidence of achieving the 24 h movement standards through the limitations of COVID-19. A total of 490 Arab Israeli parents were surveyed. An electronic cross-sectional survey was performed, including questions addressing engagement in physical activities, use of screens, and sleep duration. Throughout the COVID-19 outbreak, time spent participating in physical activity was reduced, sedentary behavior and sleep duration were increased, and the percentage of the sample who met the physical activity and sedentary behavior suggestions lessened. The percentage of participants who attained the overall 24 h movement recommendations was very low during the pandemic; school children met the guideline recommendations for physical activity and sleep duration more than preschool children, and girls spent more time in physical activity. These findings highlight the need for strategies to enhance physical activity and decrease sedentary behavior in children to prevent long-term effects of limitations imposed by COVID-19. Efforts to perceive and encourage healthy routines in Arab Israeli children in the case of pandemic limitations are expected to serve as a precedence.

## 1. Introduction

The plague of coronavirus disease 2019 (COVID-19) appeared for the first time in China and then, speedily, became distributed throughout almost all countries in the world. Consequently, by 11 March 2020, the World Health Organization (WHO) announced the COVID-19 outbreak as a global epidemic [1]. The first positive case of COVID-19 in Israel was identified on 26 February 2020. Up to the time of this study (22 January 2021), the pandemic resulted in about 600,000 positive cases and 4326 deaths in Israel [2]. To face this pandemic, Israel announced three total lockdowns (March and end of September 2020, and January 2021), locking educational facilities and substituting classroom learning with learning in small groups and, mainly, online studying activities [3,4].

Recent studies have suggested that children are at higher risk of infection with COVID-19 as part of older age groups; nevertheless, the danger of severe situations and cases of death is higher for elders and those who have other comorbidities, such as lung disease, cancer, and heart sickness [5]. Children are susceptible to serious effects from COVID-19 due to the restrictions advanced by governments, such as lockdowns and school closings, which resulted in reducing the opportunities for physical activities and changing sleep behavior [6]. 

Considering the present research, Arab Israeli citizens (almost two million; 21 percent of Israeli citizens overall) represent a linguistic and national minority group in Israel [7]. Nevertheless, in several phases of the pandemic, around 40 percent of the proven COVID-19 cases involved Arab people, and the positive cases of coronavirus tests reached nearly 12 percent among Arab residents [2]. Knowledge gained, similar to previous health emergencies, from growing data from the coronavirus pandemic have proposed that cultural or national minority peoples were more susceptible to this pandemic, as social circumstances might amplify the influence of COVID-19-related stressors on general perceptions of tension and childcare, mainly amongst minorities [8,9,10]. Arab Israeli citizens are, mostly, disadvantaged and ignored in the provision of childcaring essential services; this condition has deteriorated over the course of the coronavirus pandemic, and not many assets were directed to them [11]. Therefore, it has been anticipated that Arab Israelis—who are demographically considered to be less wealthy; have larger families, higher population mass, cultural practices, and traditions; live in outlying areas; and have a higher incidence of comorbidities—would show higher rates of infection and more challenging psychological consequences when compared to the general population [12]. Accordingly, Arab children in Israel, because of continuous school closures and the restrictions of “stay home”, may be negatively affected in terms of wellbeing through interruption of their health care, healthy behaviors, feeling of security, education, emotional and social functions, and overall psychological and mental health [5,6]. It should be noted that, in general, during the home confinement, most of the Arab parents, in cases where they worked, worked from home and, thus, were still with their children. On the other hand, if both fathers and mothers worked outside, the young children spent the time with an older sibling or older relatives, such as a grandmother. In this case, healthy behaviors such as sleep duration, physical activity, and sedentary behavior may be negatively affected.

Sleep is important for adults’ as well as children’s welfare [13,14,15]. The present conditions of staying home cause significant alterations to everyday schedules and habits. Reduction in daylight contact, along with amplified blue light contact because of huge dependence on screens and technological media, can, altogether, have undesirable consequences for sleep [16]. Consequently, the duration of sleep can affect and lessen involvement in physical activity (which has physical and psychological benefits such as improved musculoskeletal and cardiovascular strength, healthy body mass, and increased neuromuscular consciousness), the physical and mental health of children (that may maintain and improve the immune system), coping with anxiety, and development of self-esteem [17,18,19].

The previous literature has proposed that sufficient patterns of movement behaviors, e.g., physical activity (PA), sedentary behavior (SB), and sleep duration (SD), could play a critical role in maintaining health throughout the initial periods of growth and development [20,21]. Similarly, it has been pointed out that those who meet the 24 h movement recommendations (high PA/high sleep/low sedentary behavior) generally show more positive indicators relating to adiposity and cardiometabolic health in comparison with those who do not meet these recommendations [22]. Regarding the coronavirus pandemic, it has been shown that periods of applied confinement (lockdown) may lead to increase in SB and decrease in physical activity [3,23,24,25].

Recent studies performed in Brazil [26,27], Canada [6], China [28], Italy [29], Croatia [30], and Spain [26,31] have analyzed the alterations in PA, SB, and sleep duration (SD) in children and youth due to COVID-19. For example, Lopez-Gil et al. (2021) tested children’s and adolescents’ frequency of meeting the 24 h movement guidelines and changes in physical activity, sedentary behavior, and sleep duration over the period of the lockdown resultant from COVID-19, and their findings indicated that the proportion of the subjects who maintained the physical activity and screen time suggestions decreased throughout the COVID-19 restrictions in Spanish and Brazilian children, whereas sleep duration increased. Moreover, the results showed that the percentage of the subjects following the entirety of the 24 h movement recommendations was very small before the pandemic outbreak and even worse during the lockdown [26]. Moore et al. (2020), who tested the effects of coronavirus limitations on movement activities in children and youth from Canada, revealed that around 5% of children and 0.5% of youth met the combined movement behavior guidelines during COVID-19 restrictions. Additionally, they showed that children and youth had smaller PA rates, greater SB, and extended sleep during the pandemic [6]. Likewise, Zenic et al. (2020) investigated the changes in PA levels among Croatian adolescents during the COVID-19 outbreak and reported reduced physical activity levels during the COVID-19 outbreak [30].

The previous literature indicated mixed findings about physical activity, sedentary behavior, and sleep for any gender [32,33,34,35,36,37]. For example, recent research that aimed to test the impacts of COVID-19 limitations on sleep, screen time, and physical activity on children from Tunisia showed poorer sleep and greater time spent with screens in girls in comparison with boys during home confinement [32]. In contrast, other research showed that boys spent more time with screens than girls [36]. Likewise, Dana et al. (2022) demonstrated that girls had significantly higher use of smartphones both before and during the COVID-19 outbreak compared with boys; however, no gender differences were found among male and female adolescents in terms of physical activity and sleep [38]. Another study showed that, in terms of physical activity, boys had suffered more than girls from forced limitations [39].

The proportion of Arab Israeli students (about 556,000 students; 23% of the total number of Israeli students in the mandatory formal education system which spans ages 3–18 years) is higher than their proportion in the total population [40]. To our knowledge, the current study represents the first research that sheds light on alterations in meeting the 24 h movement guidelines in Israel throughout the COVID-19 lockdowns, and it may expand the evidence found in the literature about this important topic and, more importantly, examine the effect of the outbreak of COVID-19 on disadvantaged populations and ethnic minorities. Therefore, the objectives of the current research are to test the alterations in PA, SB, and SD of Arab Israeli children and the prevalence of meeting the 24 h guidelines during the lockdowns induced by COVID-19, and to asses if there are any differences between age groups and between gender groups. The following questions were addressed in the current research:

(i) Were there alterations in physical activity, sedentary behaviors, and sleep duration among Arab Israeli children during the COVID-19 outbreak, according to the perception of their parents?;
(ii) What is the prevalence of children who met the 24 h movement guidelines, in general and during the COVID-19 outbreak?;
(iii) Were there differences between boys and girls or between 3–4-year-old children and 5–13-year-old children in physical activity, sedentary behavior, sleep duration, and meeting the 24 h movement guidelines before and during the COVID-19 outbreak?

The study hypotheses are the following:

(i) We hypothesized that Arab Israeli parents would report negative changes in their children’s physical activity, sedentary behaviors, and sleep duration during the COVID-19 outbreak;
(ii) We hypothesized that the prevalence of children who met the 24 h movement guidelines in general was greater than during the COVID-19 outbreak;
(iii) We hypothesized that boys and 5–13 years old were, negatively, more affected than girls and 3–4-year-old children during the COVID-19 outbreak in terms of physical activity, sedentary behaviors, and sleep duration.

## 2. Materials and Methods

### 2.1. Participants and Setting

The present research was conducted among Arab Israeli parents via a digital survey, between 10 January and 30 January 2021, following the completion of long phases of home confinement and three general lockdowns in Israel. In the present study, non-probability sampling measures were applied to collect parents’ primary data. First of all, Israeli mothers/fathers who had at least one child aged 3–13 years old (pre-kindergarten to 6th grade) who had not been identified as having physical or psychological problems were selected, and a link to the designed Google Form of the survey was prepared by the authors and then was shared by work groups, parents’ groups, teachers’ groups, and so on. The link was supplemented with an audio clip and typed material presenting an explanation of the study, the inclusion and exclusion criteria, and its objectives, and was shared on social media applications including WhatsApp, Facebook, Instagram, and so forth. Partial forms of the questionnaire were exempted from the analyses. Overall, 518 parents sent back the filled survey to the authors and, after the exclusion of the partial answers, a total of 490 participants were held for the final analyses.

Participants did not receive any financial rewards, and information privacy was ensured. We asked for consent for participation in the study, and participants were notified that they could nullify their participation in the study whenever they wanted.

This study was executed online and followed the ethical guidelines of the U.S. Department of Health and Human Services and the human research procedure of the local College of Education (12/2020-103).

### 2.2. Data Collection Procedure

Data were collected using digital questionnaires because face-to-face interviews were prevented as part of the COVID-19 limitations.

Sociodemographic questions were included in the questionnaire (e.g., participants’ age and gender, participants’ educational level, and children’s age and gender).

#### 2.2.1. Physical Activity Measure

The question, “Generally, how many days was your child physically active for a total of at least 60 min?” served as the basis for measuring PA. This measurement has been demonstrated to have good validity and reliability [41]. Answer options were available in 1-day increments from 0 to 7 days per week. A minimum of 60 min of MVPA (moderate to vigorous physical activity) per day, seven days per week, was required to comply with the PA recommendation. In relation to the COVID-19 lockdown periods, this question was also posed.

The WHO recommends (per their 24 h movement guideline recommendations) that 3–4-year-old children should be involved in at least 180 min of physical activity (PA), participate in no more than one hour of screen time, and have 10–13 h of good-quality sleep per day [42]. For children and adolescents aged between 5 and 17 years old, it is advised to take part in at least sixty minutes of moderate to vigorous physical activity, spend a maximum of 180 min of leisure screen time, and have 8–11 h of good-quality sleep per day. The age division in this study was based on the 24 h movement guideline recommendations, and, because our study focuses on preschool and elementary school children, the second age group was limited to 13 years old.

#### 2.2.2. Sedentary Behavior Measure

Parents were required to note the amount of time their children spent engaging in various sedentary screen-based activities in order to quantify SB. For weekdays, weekends, and during the pandemic lockdown, the following questions were asked: (a) “How many hours a day does your child typically spend watching TV, videos (including YouTube or similar services), DVDs, and other screen activities?”, as well as (b) “How many hours-a-day your child typically spends playing games on a computer, games console, tablet, smartphone, or any other electronic device?”. The two questions’ weighted sum was calculated over the course of five weekdays and two weekends. The following categories apply to SB: Children and adolescents (“meeting the ST guidelines”: 1 h/d; “not meeting ST guidelines”: >2 h/d); and preschoolers (“meeting the ST guidelines”: >1 h/d) [19].

#### 2.2.3. Sleep Duration Measure

When calculating SD, parents were questioned separately about their children’s typical bedtimes and wake-up times for weekdays and weekends. Additionally, during the COVID-19 lockdown, these questions were posed. According to Lopez-Gil et al. (2021), the following formula was used to calculate the average daily SD for each participant: [(average nocturnal SD on weekdays 5) + (average nocturnal SD on weekends 2)]/7. According to the WHO’s international recommendations for the early years, responses within the ranges of 10–13 h for 3–4-year-olds and 9–11 h for 5–13-year-olds were defined as “meeting sleep guidelines”, whereas participants outside of these ranges were labeled as “not meeting sleep guidelines” [42].

### 2.3. Data Analyses

To describe the demographics of the children and their parents, descriptive statistics were performed. Descriptive data were exhibited as means (standard deviations) for continuous variables and as numbers (percentages) for categorical variables. The percentage of children meeting the 24 h guidelines was calculated. Differences between age groups and gender groups in sociodemographic and anthropometrical information were evaluated using the chi-square (χ^2^) test for categorical variables and Cramer’s *V* effect size for chi-square.

In addition, a repeated measures analysis of variance (rm-ANOVA) was performed to compare groups with respect to sleep duration, physical activity, and sedentary behavior before and during the COVID-19 outbreak. If rm-ANOVA produced a significant interaction, independent sample *t*-test analysis and Cohen’s *d* effect size for *t*-test were used to understand the basis of the interaction.

Differences in the achievement of the 24 h physical activity recommendations and individual component suggestions were examined using the independent McNemar’s test (categorical variables). A *p*-value of 0.05 indicated statistical significance.

## 3. Results

Table 1 presents the sociodemographic characteristics of parents and children.

The results showed no significant differences between the 3–4-year-olds and 5–13-year-olds, nor between boys and girls in any of the sociodemographic variables, except the education level of the parents.

### 3.1. Physical Activity, Sedentary Behavior, and Sleep Duration Pre- and during COVID-19 Outbreak

Table 2 shows the physical activity, sedentary behavior, and sleep duration in children before and during the COVID-19 outbreak among the total sample, as well as divided by age group and gender, by means and standard deviations.

Overall, the results show a significant decrease in PA (*F*(1, 489) = 640.92, *p* < 0.001, *η*^2^ = 0.57), a significant increase in SB (*F*(1, 489) = 358.25, *p* < 0.001, *η*^2^ = 0.42), and a significant increase in SD (*F*(1, 489) = 195.34, *p* < 0.001, *η*^2^ = 0.29) during the COVID-19 outbreak compared with the period before.

### 3.2. Age Group Differences

**Figure 1:**
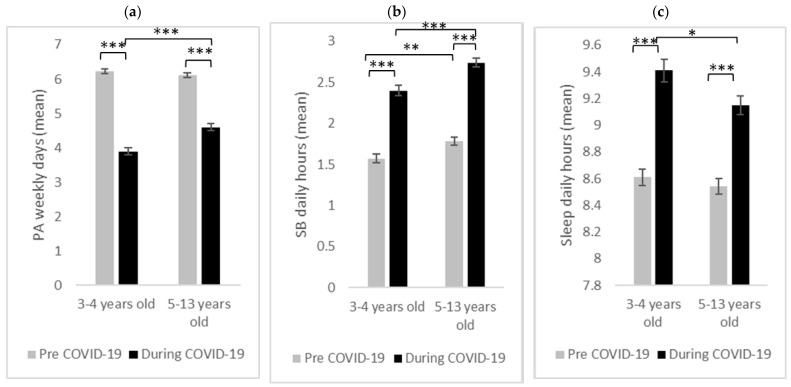
Children’s (**a**) physical activity (PA), (**b**) sedentary behavior (SB), and (**c**) sleep duration before and during the COVID-19 outbreak, as reported by the parents, divided according to the age group. * *p* < 0.05, ** *p* < 0.005, *** *p* < 0.001.

Figure 1 shows the mean values (aggregated and stratified by age group) pre- and during the COVID-19 outbreak for (a) physical activity, (b) sedentary behavior, and (c) sleep duration abundance. 

#### 3.2.1. Physical Activity

As reported by the parents, there was a significant decline in engagement in PA during the COVID-19 outbreak (*F*(1, 488) = 679.64, *p* < 0.001, *η*^2^ = 0.58). In addition, there was a significant main effect of age group (*F*(1, 488) = 7.95, *p* = 0.005, *η*^2^ = 0.01) that was modulated by a significant interaction between physical activity and age group (*F*(1, 488) = 31.46, *p* < 0.001, *η*^2^ = 0.06). The interaction emerged because there were no significant differences between the two age groups before the COVID-19 outbreak (*t*(488) = 1.09, *p* = 0.278, *d* = 0.10), while there were significant differences during the COVID-19 outbreak (*t*(488) = 4.72, *p* < 0.001, *d* = 0.43), indicating that the children in the 3–4-year-old group engaged in fewer days of sufficient PA than the 5–13-year-old children during the COVID-19 outbreak (Figure 1a).

#### 3.2.2. Sedentary Behavior

There was a significant increase in SB during the COVID-19 outbreak (*F*(1, 488) = 358.93, *p* < 0.001, *η*^2^ = 0.42). In addition, there was a significant main effect of age group (*F*(1, 488) = 20.57, *p* < 0.001, *η*^2^ = 0.04), showing that the 5–13-year-old age group spent more time with screens. However, there was no significant interaction between sedentary behavior and age group (*F*(1, 488) = 1.67, *p* = 0.196, *η*^2^ = 0.00). Notably, there were significant differences between the two age groups before (*t*(488) = 2.94, *p* = 0.003, *d* = 0.27) and during (*t*(488) = 4.22, *p* < 0.001, *d* = 0.38) the COVID-19 outbreak, indicating that the older age group spent more time per day with a screen than the 3–4-year-old children before and during the COVID-19 outbreak (Figure 1b).

#### 3.2.3. Sleep Duration

The results indicate that there was a significant increase in the SD during the COVID-19 outbreak (*F*(1, 488) = 196.21, *p* < 0.001, *η*^2^ = 0.29). In addition, there was a significant main effect of age group (*F*(1, 488) = 3.80, *p* = 0.050, *η*^2^ = 0.01) that was modulated by a significant interaction between sleep duration and age group (*F*(1, 488) = 3.80, *p* = 0.050, *η*^2^ = 0.01). The source of the interaction was that there were no significant differences between the two age groups before the COVID-19 outbreak (*t*(488) = 0.77, *p* = 0.441, *d* = 0.07), whereas there were significant differences between the two age groups during the COVID-19 outbreak (*t*(488) = 2.63, *p* = 0.019, *d* = 0.24), demonstrating that the 3–4-year-old age group slept more hours per day than the 5–13-year-old age group during the COVID-19 outbreak (Figure 1c).

### 3.3. Gender Differences

**Figure 2:**
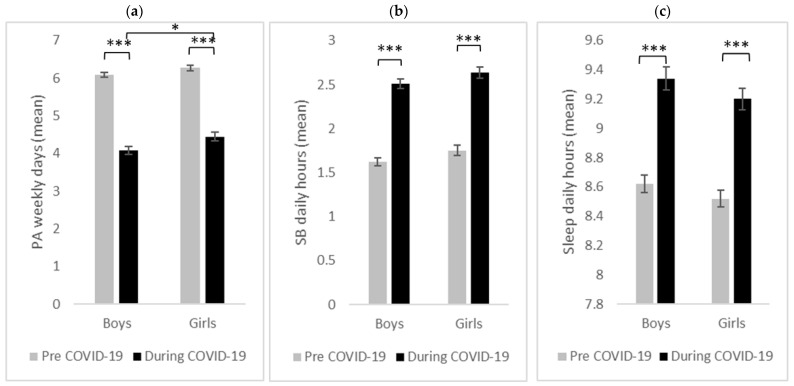
Children’s (**a**) physical activity (PA), (**b**) sedentary behavior (SB), and (**c**) sleep duration before and during the COVID-19 outbreak, as reported by the parents, according to the gender. * *p* < 0.05, *** *p* < 0.001.

Figure 2 illustrates the mean (aggregated and stratified by gender) before and during the COVID-19 outbreak for the amounts of (a) PA, (b) SB, and (c) SD.

#### 3.3.1. Physical Activity

Overall, there was a significant decline in engaging in PA during the COVID-19 outbreak in comparison to the period before (*F*(1, 488) = 626.20, *p* < 0.001, *η*^2^ = 0.56), as well as a significant main effect of gender (*F*(1, 488) = 6.43, *p* = 0.012, *η*^2^ = 0.01), indicating that, overall, boys engaged fewer days per week in PA than girls. Independent sample *t*-test showed that there were no significant differences between the two groups before COVID-19 (*t*(488) = 1.73, *p* = 0.085, *d* = 0.16), while there were significant differences during the COVID-19 outbreak (*t*(488) = 2.36, *p* = 0.019, *d* = 0.21). However, there was no significant interaction between physical activity and gender (*F*(1, 488) = 1.47, *p* = 0.225, *η*^2^ = 0.00). Thus, boys (*F*(1, 272) = 424.77, *p* < 0.001, *η*^2^ = 0.61) and girls (*F*(1, 217) = 229.77, *p* < 0.001, *η*^2^ = 0.52) showed a significant decrease in performing PA during the COVID-19 outbreak (Figure 2a).

#### 3.3.2. Sedentary Behavior

The analyses show that there was a significant increase in engagement in SB during the COVID-19 outbreak in comparison to the period before (*F*(1, 488) = 353.19, *p* < 0.001, *η*^2^ = 0.42). In addition, there was a significant main effect of gender (*F*(1, 488) = 4.37, *p* = 0.037, *η*^2^ = 0.01), showing that, overall, girls spent more time with screens. However, there was no significant interaction between sedentary behavior and gender (*F*(1, 488) = 0.01, *p* = 0.936, *η*^2^ = 0.00). Notably, there were no significant differences between the two genders before (*t*(488) = 1.67, *p* = 0.090, *d* = 0.15) and during (*t*(488) = 1.65, *p* = 0.101, *d* = 0.15) the COVID-19 outbreak (Figure 2b).

#### 3.3.3. Sleep Duration

There was a significant increase in the SD during the COVID-19 outbreak (*F*(1, 488) = 191.45, *p* < 0.001, *η*^2^ = 0.28). However, there was no significant main effect of gender (*F*(1, 488) = 1.98, *p* = 0.161, *η*^2^ = 0.00) as well as no significant interaction between sleep duration and gender (*F*(1, 488) = 0.10, *p* = 0.758, *η*^2^ = 0.00) (Figure 2c).

### 3.4. Proportions Meeting the 24 h Movement Guidelines

Table 3 shows the proportion of children meeting the 24 h movement guidelines (%).

Overall, the proportion of participants who met the PA and SB guidelines decreased during the COVID-19 outbreak, while the SD increased significantly (*p* < 0.001 for the three measures). Notably, meeting the 24 h movement guidelines deteriorated significantly during the COVID-19 outbreak (*p* < 0.001).

#### 3.4.1. Age Group Comparison

The proportion of participants who met the PA and SB guidelines decreased during the COVID-19 outbreak, in both the 3–4-year-old and 5–13-year-old samples (*p* < 0.001 for both age groups), while the SD increased (*p* < 0.001) (Table 3). The proportion who met the 24 h movement guidelines decreased in both age groups (*p* = 0.001 for the 3–4-year-old group and *p* < 0.001 for the 5–13-year-old group). The 5–13-year-old group met the 24 h movement guidelines before the pandemic more than the younger age group in SB and SD. Moreover, the older age group tended to meet the 24 h movement guidelines during the COVID-19 outbreak more than 3–4-year-old age group, in both the PA measure and the SD measure. Overall, the 5–13-year-old group met the 24 h movement guidelines more than the 3–4-year-old group before the COVID-19 outbreak, while there were no significant differences during the pandemic.

#### 3.4.2. Gender Comparison

The proportion of participants who met the PA and SB guidelines decreased during the COVID-19 outbreak in boys and girls (*p* < 0.001 for both), while SD increased for boys and girls (*p* < 0.001). Notably, girls met the 24 h movement guidelines before the pandemic more than boys for SB while the boys outperformed the girls in SD. However, there was no significant difference between the proportions of boys and girls during the COVID-19 outbreak in meeting any of the three measures of the 24 h movement guidelines.

The arrows in the table represent the changes in the percentage of children meeting the 24 h movement guidelines for physical activity, sedentary behavior, sleep duration, and the combined guidelines.

## 4. Discussion

The present research aimed to test alterations in physical activity, sedentary behavior, and sleep duration in children, and the proportions who met the 24 h movement guidelines before and during the coronavirus outbreak, concerning age and gender differences, in a sample from the Arab Israeli population.

### 4.1. Physical Activity

The findings of the current study are in line with those of others who reported a decline in the time spent participating in PA [3,5,6,26,31,43,44,45] and in meeting PA recommendations [6,26,27,28,46]. The PA decline occurred in different age groups and genders. The results reveal a serious and risky descending trend in regular PA levels among children during the COVID-19 outbreak [6,26,28,46]. Social limitations involving digital and remote learning and home confinement recommendations have made it difficult for children to participate in physical education lessons, sports, or other forms of school-related PA; therefore, a decrease in outdoor play could result in reduced PA [26,47].

The findings of the present study show that, during the COVID-19, more participants from the older age group (5–13 years old children) met the PA guidelines, and the 3–4-year-olds saw a greater decrease in PA. These results contrast with Lopez-Gil et al. (2021) [26], who found that, during COVID-19 lockdowns, Spanish and Brazilian preschoolers met the PA guideline recommendations. Likewise, Aguilar-Farias et al. (2021) [48] found that Chilean 4-year-old children met the PA guidelines more than 5-year-old children. The different patterns in results can be explained by the different sample sizes (3157 participants in Aguilar-Farias et al. and 1099 participants in Lopez-Gil et al.) or by cultural differences, reflecting the fact that Arabs in Israel, as an ethnic and cultural minority, are usually disadvantaged in child protective service resources and facilities. This occurs especially in the early ages such as preschool stage, and this situation was worsened during COVID-19 with fewer resources targeted towards them [11,12].

Girls tended to meet the PA guidelines more than boys before the COVID-19 outbreak. However, this advantage disappeared during the outbreak; this can be explained by the fact that the girls replaced their PA with engagement in more screen time and social media during COVID-19 [6].

### 4.2. Sedentary Behavior

The outcomes also show an increase in the SB of preschoolers, children, boys, and girls during the COVID-19 outbreak. These outcomes are in line with other research executed pertaining to information about the COVID-19 outbreak and lockdowns [6,26,30,44,45,46]. These results could be described by the circumstances of the COVID-19 outbreak and home confinement restrictions, which could accelerate the “displacement theory” [26]. This theory suggests that screen time can replace time spent carrying out PA. Children were less active, with greater weight indicators by age 8 and 10, and children at age 6 watching less television [49]. An additional possible reason for these findings may be the inability of parents to restrict SB in their children [46]. Studies have shown a correlation between children’s screen time and reduced psychological well-being, with excessive screen time by children associated with psychological aspects such as reduced self-control, emotional lability, and symptoms of depression. The cost of use is emphasized [50,51]. Still, it is important to recognize that this is a health emergency at this time, and activities should be restructured to render schedules as close to normal routines as possible. This finding is alarming, and approaches to reduce this excessive behavior need to be found and applied. Therefore, keeping a daily routine is crucial [52]; where workable, parents should consider replacing sedentary leisure with more active leisure hobbies.

In line with Moore et al. (2020) [6] and Aguilar-Farias et al. (2021) [48], there were no significant differences between boys and girls in terms of engaging in SB nor in meeting the SB guidelines pre- and during the COVID-19 outbreak. Importantly, the findings show that 5–13-year-old group tended to engage more in SB pre- and during the outbreak of the pandemic than 3–4-year-olds, and more participants from the 5–13-year-olds met the guideline recommendation of SB before the outbreak; however, there were no significant differences between the two age groups in meeting the SB guidelines during the outbreak of coronavirus. This can be explained by the fact that preschoolers need more play space than others and are more likely to have access to screen-based devices. It can also be partly explained by the fact that more educated parents have to work from home. Parents should aim to reduce screen use to entertain young children while caregivers work from home [36].

### 4.3. Sleep Duration

The findings of the present study indicate that not only the SD, but also the percentage of participants who met the sleep recommendations increased during the COVID-19 crisis in all groups. Those findings are similar to Lopez-Gil et al. (2020) [23] who reported an increase in SD and meeting SD guidelines among Spanish and Brazilian children. Likewise, Moore et al. (2020) [4] reported an increase in the SD of Canadian children and adolescents, as was described by López-Bueno et al. (2020) [28] in a Spanish sample. A similar pattern of results was reported by Francisco et al. (2020) [32] in a sample of children from Spain, Italy, and Portugal. This increase in SD might be supported by the absence of commuting to school, allowing children to spend more time asleep. Nevertheless, Zreik, Asraf, Haimov, and Tikotzky (2021) [53] found no change in SD of Israeli children during the COVID-19 pandemic in comparison with the previous period. This outcome pattern can be explained by the different age of the children in their research (0.5 to 6 years old) compared to ours. Thus, as reported by many studies about SD, the results of the current study showed a positive change in children’s behaviors during the pandemic outbreak. However, our study did not ask about the sleep quality of the children. If this increase in sleep hours is associated with poor sleep quality, it could be problematic; this concept should be investigated in future research. In line with other studies, there were no significant differences between girls and boys before and during the pandemic crisis [6,48]. Notably, the 3–4-year-old group tended to sleep more hours per day during the COVID-19 outbreak than 5–13-year-old children; however, more participants from the 5–13-year-old group met the sleep recommendations guidelines. Notably, 3–4-year-old children are recommended to sleep 10–13 h per day, while the 5–15-year-olds are recommended to sleep 8–11 h per day [42]. Thus, the proportion who met the sleep recommendations guideline increased in both age groups.

The findings of the current study should be taken into consideration to advance healthful daily life strategies and practices for children during and after the restrictions of the pandemic. Consequently, it is suggested to increase PA and reduce sedentary behavior during the lockdown, in order to improve healthy behavior in children. Parents, teachers, health representatives, and authorities need to be aware of this situation and should make efforts to enhance practical strategies and interventions to improve PA levels and prevent negative health-related behaviors.

### 4.4. Limitations and Implications for Future Studies

To our knowledge, the current study is one of the first Israeli studies that provides information about the impacts of the COVID-19 outbreak on children’s healthy behaviors and movement guidelines. Moreover, it is the first study that focuses on the Arab minority in Israel in terms of healthy behaviors and 24 h movement guidelines. In addition, this study contributes to the existing literature that highlights the negative effects of COVID-19 restrictions on children.

The current research has the following limitations. This cross-sectional kind of survey cannot make conclusions as to cause–effect relationships, and participation through social network groups might yield bias. Considering the exceptional situation of the pandemic, face-to-face meetings were prevented, but, compared to in-person meetings, digital self-reporting has some limitations. However, it should be considered that an online survey, such as Google Forms, is the most rapid and reliable procedure to gather data in a limited period and, in particular, if social interactions are prevented (e.g., due to lockdown) [54].

It would be more efficient to enroll a greater sample size, but it was hard to gather larger samples on a wider scale in the existing situation. Finally, because the study consists of Arabic-speaking parents only, the results may not be generalizable to populations other than Arab Israelis. Nonetheless, the outcomes of this research confirm results from questionnaire studies on children from around the world, including Italy and Spain [29,55], China [56], Australia [57], Canada [4], and Tunisia [32]. Accordingly, it is probable that results in the present study might be generalizable to those in other parts of the world who experienced pandemic-related limitations.

Future research should compare the outcomes of COVID-19 between Arab children and Jewish children in Israel, to study the possibility of ethnic and social effects. Further research should also assess potential mediators related to parents or the home environment (e.g., parental stress, hours worked from home) that may impair responsiveness. This would help to meet the needs of parents in caring for their children. 

Self-reporting by parents is not an objective report of sleep, physical activity, or sedentary behavior. Therefore, future research should use more valid and objective measures.

## 5. Conclusions

This study has indicated that all movement behaviors among Arab Israeli children were altered during the outbreak of the COVID-19 pandemic. The current study suggests that extended periods of social isolation, homestays, and school closure might play a large threat in expanding unwise habits, such as the excessive use of screens, and decreasing vital habits, such as physical activities. Consequently, the findings suggest that schools should encourage children and parents to maintain healthy routines with an adequate sleep cycle and physical activity, and media should be used to promote exercise.

Thus, classroom programs should be contained in school health guidelines as an integrative approach, in collaboration with educative sponsors, to re-establish the suggested level of physical activity and decrease the growth in sedentary behavior [39]. These considerations prove the necessity of establishing educational networks (family, school, sport, and recreational environment) which are allied with each other to focus on the problem of child sedentarism.

During lockdowns, people should be authorized to take part in exercise in an open space. Organizations, systems, institutions such as the Ministry of Health and Ministry of Education, the health system, and the education system, together with employees, teachers, and educators, should organize demonstrations of a variety of practices and strategies for the population via mass and social media.

Government institutions and schools should develop programs designed to contribute to the psychological health and to foster sleep and movement behavior in children that have been critically affected, mainly, in ethnic minorities or at-risk residents such as Arab Israeli children.

## Figures and Tables

**Table 1:** Sociodemographic characteristics of parents and children, and comparisons between children’s age and gender groups.

	Total (*n* = 490)	3–4 Years Old (*n* = 246)	5–13 Years Old (*n* = 244)	Test ^a^ (Effect Size ^b^)	Males (*n* = 273)	Females (*n* = 217)	Test ^a^ (Effect size ^b^)
Parents							
Female [N(%)]	418 (85.3)	214 (87)	204 (83.6)	1.12 (0.05)	229 (83.9)	189 (87.1)	0.99 (0.05)
Age [M]	38.77	37.01	40.56	1.05 (0.13)	38.60	38.98	1.01 (0.12)
Education [N(%)]				**20.55 (0.21)**			**36.50 (0.27)**
≤Secondary BA degree ≥MA degree	181 (36.9) 174(35.5) 135 (27.6)	111 (45.1) 87 (35.3) 48 (19.5)	70 (28.6) 87 (35.7) 87 (35.7)		126 (46.1) 99 (36.2) 48 (17.5)	55 (25.3) 75 (34.6) 87 (40.1)	
Working (yes)	372 (75.9)	182 (74)	190 (77.9)	1.01 (0.05)	208 (76.2)	164 (75.6)	0.03(0)
Was a member of your family diagnosed with COVID-19? (yes)	191 (39)	99 (40.2)	92 (37.7)	1.50 (0.08)	105 (38.5)	86 (39.6)	0.39 (0.04)
Was a member of your family in isolation? (yes)	354 (72.2)	180 (73.2)	174 (71.3)	0.15 (0.02)	209 (76.6)	154 (71)	1.01 (0.06)
**Children**							
Male [N(%)]	273 (55.7)	137 (55.7)	136 (55.7)	0.01 (0)			
Age [M (SD)]		3.18 (0.49)	7.25 (0.84)				
Age Level							0.64 (0.05)
3–4 years old	246 (50.2)				116 (47.1)	130 (52.8)	
Diagnosed with COVID-19	63 (12.9)	35 (14.2)	28 (11.5)	0.44 (0.04)	37 (13.5)	26 (12)	0.39 (0.04)
Asked to be in isolation	243 (49.6)	122 (49.5)	121 (49.6)	0.01 (0)	136 (49.8)	107 (49.3)	0.02 (0.01)

M, mean; SD, standard deviation. ^a^ Cross-table (*X*^2^) for categorical variables. ^b^ Cramer’s V for multi-categorical variables. The bold type denotes significant values.

**Table 2:** Physical activity, sedentary behavior, and sleep duration values (by means and standard deviations) in children’s age and gender groups before and during the COVID-19 outbreak.

Measure	Total (*n* = 490)		3–4 Years Old (*n* = 246)		5–13 Years Old (*n* = 244)		Boys (*n* = 273)		Girls (*n* = 217)	
	Pre *M* (*SD*)	During *M* (*SD*)	Pre *M* (*SD*)	During *M* (*SD*)	Pre *M* (*SD*)	During *M* (*SD*)	Pre *M* (*SD*)	During *M* (*SD*)	Pre *M* (*SD*)	During *M* (*SD*)
Physical activity (days per week)	6.17 (1.50)	4.24 (1.71)	6.23 (1.13)	3.89 (1.65)	6.11 (1.67)	4.60 (1.71)	6.09 (1.17)	4.08 (1.67)	6.27 (1.12)	4.45 (1.74)
Sedentary behavior (hours per day)	1.68 (0.82)	2.57 (0.90)	1.57 (0.85)	2.40 (0.95)	1.78 (0.78)	2.74 (0.81)	1.62 (0.79)	2.51 (0.88)	1.75 (0.87)	2.64 (0.94)
Sleep duration (hours per day)	8.57 (0.92)	9.28 (1.21)	8.61 (0.95)	9.41 (1.30)	8.54 (0.91)	9.15 (1.11)	8.62 (0.99)	9.34 (1.32)	8.52 (0.84)	9.20 (1.07)

Pre = before the COVID-19 outbreak. During = during COVID-19 outbreak.

**Table 3:** Proportion of children meeting guidelines.

	Total % (*n* = 490)	3–4 Years Old % (*n* = 246)	5–13 Years Old % (*n* = 244)	*t* (*p*, *d*)	Boys % (*n* = 273)	Girls % (*n* = 217)	*t* (*p*, *d*)
Before COVID-19 Outbreak							
PA	59.2	61.8	56.6	1.18 (0.240, 0.11)	54.6	65	**−2.33 (0.020, 0.21)**
SB	60	53.2	66.8	**−3.09 (0.002, 0.28)**	61.5	58.1	0.78 (0.437, 0.07)
SD	36.1	28	44.3	**−3.78 (<0.001, 0.34)**	39.9	31.3	**1.97 (0.049, 0.18)**
24 h combined	12	7.7	16.4	**−2.97 (0.003, 0.27)**	12.8	11.1	0.59 (0.533, 0.05)
**During COVID-19 Outbreak**							
PA	20.6 ↓	16.3 ↓	25 ↓	**−2.40 (0.017, 0.22)**	17.9 ↓	24 ↓	1.64 (0.102, 0.15)
SB	20.8 ↓	21.5 ↓	20.1 ↓	0.40 (0.691, 0.04)	19.4 ↓	22.6 ↓	−0.86 (0.392, 0.08)
SD	56.1 ↑	50.8 ↑	61.5 ↑	**−2.38 (0.017, 0.22**)	65.4 ↑	55.8 ↑	0.14 (0.886, 0.01)
24 h combined	3.1 ↓	2.4 ↓	3.7 ↓	−0.80 (0.423, 0.07)	2.2 ↓	4.1 ↓	−1.24 (0.214, 0.11)

PA = physical activity, SB = sedentary behavior, SD = sleep duration, 24 h combined = meeting the 24 h movement guidelines. The bold type denotes significant values. ↑ = significant increase (*p* < 0.001), ↓ = significant decrease (*p* < 0.001).

## Data Availability

The data presented in this study are available on request from the corresponding author.
